# Fabrication of Cellulose Film with Enhanced Mechanical Properties in Ionic Liquid 1-Allyl-3-methylimidaxolium Chloride (AmimCl)

**DOI:** 10.3390/ma6041270

**Published:** 2013-03-26

**Authors:** Jinhui Pang, Xin Liu, Xueming Zhang, Yuying Wu, Runcang Sun

**Affiliations:** 1 Beijing Key Laboratory of Lignocellulosic Chemistry, Beijing Forestry University, Beijing 100083, China; E-Mails: pangjinhui1223@126.com (J.P.); meteor1005@hotmail.com (X.L.); wuyuying-1980@163.com (Y.W.); 2 State Key Laboratory of Pulp and Paper Engineering, South China University of Technology, Guangzhou 510640, China

**Keywords:** cellulose film, ionic liquid, mechanical property, thermal stability

## Abstract

More and more attention has been paid to environmentally friendly bio-based renewable materials as the substitution of fossil-based materials, due to the increasing environmental concerns. In this study, regenerated cellulose films with enhanced mechanical property were prepared via incorporating different plasticizers using ionic liquid 1-allyl-3-methylimidazolium chloride (AmimCl) as the solvent. The characteristics of the cellulose films were investigated by scanning electron microscopy (SEM), atomic force microscopy (AFM), thermal analysis (TG), X-ray diffraction (XRD), ^13^C Solid-state cross-polarization/magic angle spinning nuclear magnetic resonance (CP/MAS NMR) and tensile testing. The results showed that the cellulose films exhibited a homogeneous and smooth surface structure. It was noted that the thermal stability of the regenerated cellulose film plasticized with glycerol was increased compared with other regenerated cellulose films. Furthermore, the incorporation of plasticizers dramatically strengthened the tensile strength and improved the hydrophobicity of cellulose films, as compared to the control sample. Therefore, these notable results exhibited the potential utilization in producing environmentally friendly cellulose films with high performance properties.

## 1. Introduction

Natural biopolymers from renewable resources have attracted much interest as alternatives for petroleum-based polymers, due to the increasing environmental concerns and decreasing fossil resources [1]. Cellulose, the most abundant natural biopolymer on earth, is considered as one of the most promising polymeric resource. It has many advantages, such as low cost, biocompatibility and biodegradability [2], which not only allow it to be used in furniture, clothing, packaging, paper and medical products in our daily life, but also give the potential in numerous applications as bio-based materials, such as fibers, films, food casings and membranes [3,4,5]. Therefore, effective utilization of cellulose will reduce the consumption of our limited fossil resources so as to protect the environment. 

In recent years, considerable attention has been directed towards biodegradable cellulose-based materials, due to the serious “white pollution” made from the non-biodegradable plastic film [6]. It is known that dissolution of cellulose is of fundamental importance for its processing and chemical derivatization [7]. However, the utilization and derivatization of cellulose are difficult, because cellulose is neither meltable nor soluble in water or common solvents, due to its partially crystalline structure and hydrogen bonds [8]. In a long time, the processability and utilization of cellulose have been limited by the lack of a suitable solvent for cellulose regeneration process. Over the past decades, several cellulose solvent systems were available for dissolving cellulose, such as the LiCl/*N*,*N*-dimethylacetamide system [9], *N*-methylmorpholine-*N*-oxide (NMMO) [10] and NaOH-water with or without additives [11]. However, these conventional cellulose solvent systems had disadvantages, such as limited dissolving capability, toxicity, high cost, solvents recovery, uncontrollable side reaction and instability during cellulose processing and/or derivatization [12]. In recent years, ionic liquids, as a new type of environmentally friendly “green solvent”, have excellent characteristics, such as high thermal stability and electrochemical stability [13,14], lower viscosity, non-flammability and immeasurable low vapor pressure [12]. In addition, ionic liquids exhibited outstanding dissolving capability for cellulose [15], which would broaden the comprehensive utilization for cellulose [16]. The dissolution and regeneration of cellulose in different ionic liquids have been reported in recent studies [17,18]. Xiong *et al.* [18] prepared the cellulose/polycaprolactone (PCL) blend films using 1-butyl-3-methylimidazolium chloride (BMIM[Cl]) as the solvent. The results showed that the blends exhibited significant enhancement of thermal stability compared to the regenerated cellulose. In addition, homogeneous acetylation of cellulose and preparation of cellulose/multiwalled-carbon-nanotube composite fibers in 1-allyl-3-methylimidazolium chloride (AmimCl) were investigated by Zhang and coworkers [19,20,21]. The results indicated that AmimCl was a highly efficient cellulose solvent, and the regenerated cellulose exhibited good mechanical properties. Recently, Mahadeva [22] studied the effect of 1-butyl-3-methylimidazolium bis (trifluoromethylsulfonyl)imide (BMITFSI) blending on the electrical properties of cellulose. The results showed that an eight-fold increase in electromechanical properties of the BMITFSI-loaded cellulose actuator was achieved upon dispersion of BMITFSI in the cellulose matrix. Moreover, residual ionic liquid on the thermal stability and the electromechanical behavior of cellulose regenerated from 1-ethyl-3-methylimidazolium acetate was also investigated by Mahadeva and Kim [23], and they found that there was still a small amount of residual ionic liquid entrapped in the films after the regeneration process.

Although some research has been carried out to prepare the celluloses-based biodegradable films [24,25], the brittle characteristic, poor mechanical behavior and water sensitivity are still the key problems that restrict their uses in a wide range of applications. To improve cellulose-based polymer chemical or physical properties, plasticization is a very simple, but useful method. Glycerol has been investigated as a plasticizer for starch-poly (vinyl alcohol) blends film, and it was found that glycerol content was important to predict the ultimate tensile strength [26]. In addition, incorporation of biodegradable reinforcements, such as xylitol, sorbitol and carboxymethyl cellulose (CMC), into hemicelluloses and other polymers has already been proven to be an important strategy for obtaining nanocomposites with high mechanical performance [27,28]. In this study, we attempted to improve the mechanical properties and hydrophobicity of regenerated cellulose films by incorporating of plasticizers of sorbitol, glycerol and carboxymethyl cellulose (CMC). Ionic liquid solvent, 1-allyl-3-methylimidazolium chloride (AmimCl), was used to prepare plasticizers/cellulose blend films, due to its powerful dissolubility for cellulose and relatively lower price. The structure and properties of regenerated cellulose films were investigated to evaluate the desirability of their applications in the packaging and functional materials fields.

## 2. Results and Discussion

### 2.1. Topography of Films

**Figure 1:**
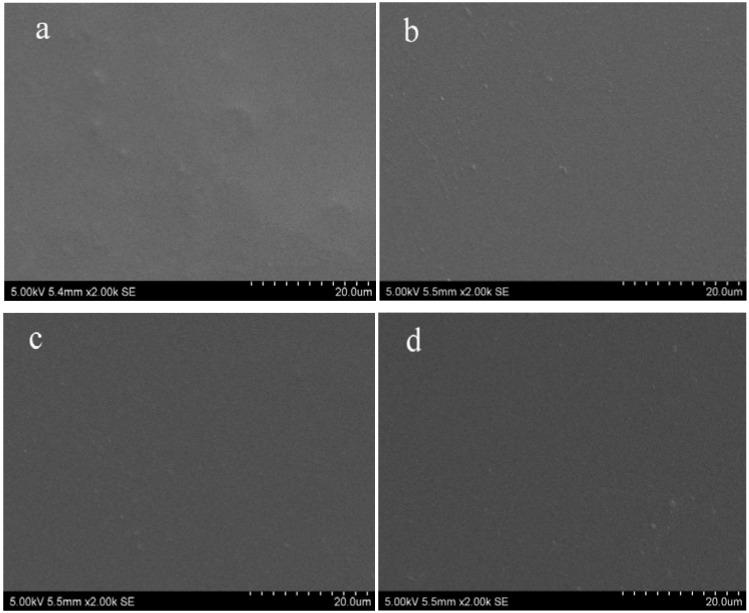
Scanning electron microscope (SEM) images of films: (**a**) control sample; (**b**) cellulose film plasticized with sorbitol; (**c**) cellulose film plasticized with glycerol; (**d**) cellulose film plasticized with carboxymethyl cellulose (CMC).

**Figure 2:**
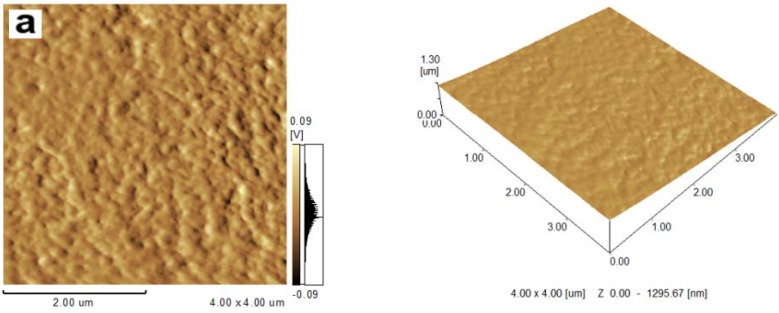

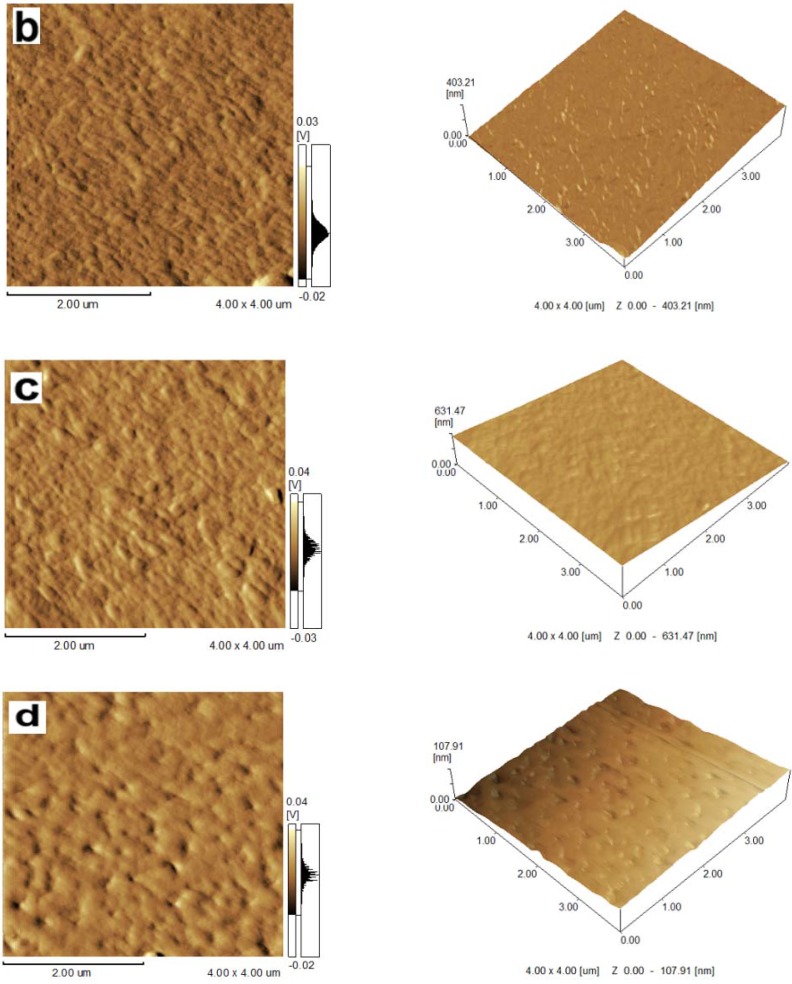
Atomic force microscopy (AFM) images of films: (**a**) control sample; (**b**) cellulose film plasticized with sorbitol; (**c**) cellulose film plasticized with glycerol; (**d**) cellulose film plasticized with CMC.

The morphology of cellulose films was analyzed by scanning electron microscopy (SEM) and atomic force microscopy (AFM), and images are shown in Figure 1 and Figure 2. It was shown from SEM views that all regenerated cellulose films exhibited smooth and homogeneous morphology with no sign of phase separation, which indicated the complete miscibility between cellulose and plasticizers. In addition, the cellulose film blended with plasticizers displayed smoother and more homogeneous topography compared with the controlled cellulose films. The higher resolution AFM images of the regenerated cellulose films are observed in Figure 2. The micrographs proved that the surfaces of regenerated cellulose films displayed homogeneous structures with typical granular morphology. It was also noted that the *z*-axis scale of the control sample was bigger than that in the plasticized cellulose films, which meant that the roughness of the plasticized cellulose films was increased in the presence of plasticizers.

### 2.2. Water Contact Angle 

The interaction of the regenerated cellulose films with water was investigated by contact angle, and the data are shown in Table 1. It is known that cellulose and plasticizers (sorbitol, glycerol and CMC) are highly hydrophilic in nature. However, the regenerated cellulose films became more hydrophobic with the presence of plasticizers. It was observed that the contact angles of the plasticized cellulose films were increased by 63.2%, 104.4% and 105.2% in the presence of sorbitol, glycerol and CMC, respectively. The increased hydrophobic nature of the regenerated cellulose films might be correlated with their morphology. It was noted from Figure 1 that regenerated cellulose films exhibited smooth and homogeneous structures. This lowers the interfacial free energy, thereby preventing water to penetrate into the films, thus increasing the contact angle. Moreover, it might be assumed that the inter- and intra-molecular hydrogen bonding within the cellulose hydroxyl groups and plasticizers also contributed to the reduction in surface free energy; thereby, increasing contact angle value of these blends [29]. The increase of contact angle in plasticized cellulose films is beneficial for the utilization in packaging.

**Table 1:** Water contact angle values of cellulose films.

Samples	Contact angle (degree/◦)
Control sample	36.4
Cellulose film plasticized with sorbitol	59.4
Cellulose film plasticized with glycerol	74.4
Cellulose film plasticized with CMC	74.7

### 2.3. FTIR Spectra

**Figure 3:**
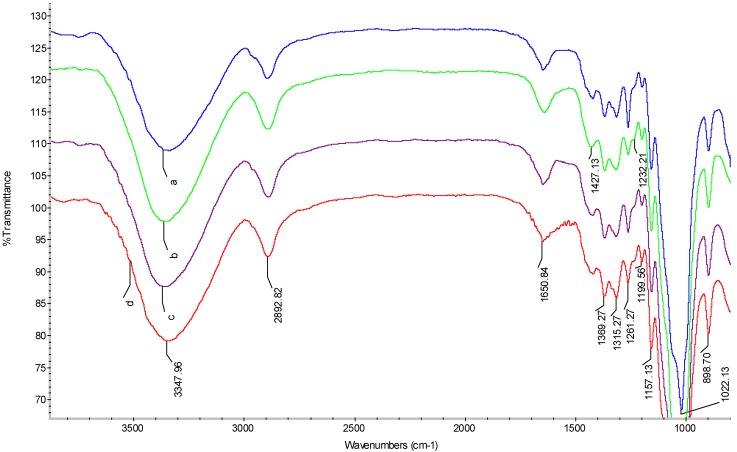
The FTIR spectra of cellulose films: (**a**) control sample; (**b**) cellulose film plasticized with sorbitol; (**c**) cellulose film plasticized with glycerol; (**d**) cellulose film plasticized with CMC.

The FTIR spectra of the controlled cellulose film (spectrum a) and the regenerated films prepared in the presence of sorbitol (spectrum b), glycerol (spectrum c) and CMC (spectrum d) are shown in Figure 3. The peak at 3347 cm^−1^ is originated from the stretching of –OH groups, and the peak at 2892 cm^−1^ is assigned to CH-stretching. The band at 1650 cm^−1^ is due to water in the amorphous region [30]. The strong band at 1022 cm^−1^ is attributed to the characteristic C–O–C stretching. The band at 1427 cm^−1^ in all spectra indicated that all samples contained a mixture of crystallized cellulose II and amorphous cellulose [31]. Bands due to C–O antisymmetric bridge stretching and C–O–C pyranose ring skeletal vibration were detected at 1157 cm^−1^. A small sharp band at 898 cm^−1^ represents the glycosidic C_1_–H deformation with a ring vibration contribution, which is indicative of β-glycosidic linkages between the sugar units. Moreover, the intensity of this band was relatively higher, as compared with cellulose I [32], which corroborated with the transition from cellulose I to II. The bands at 1369, 1315 and 1157 cm^−1^ are assigned to CH-stretching, CH_2_ wagging and C–O stretching in cellulose II, respectively [33]. Therefore, it could be concluded that the regenerated cellulose films were partially transferred from cellulose I to cellulose II without any derivatization during the dissolution and regeneration processes. 

### 2.4. X-ray Diffraction

**Figure 4:**
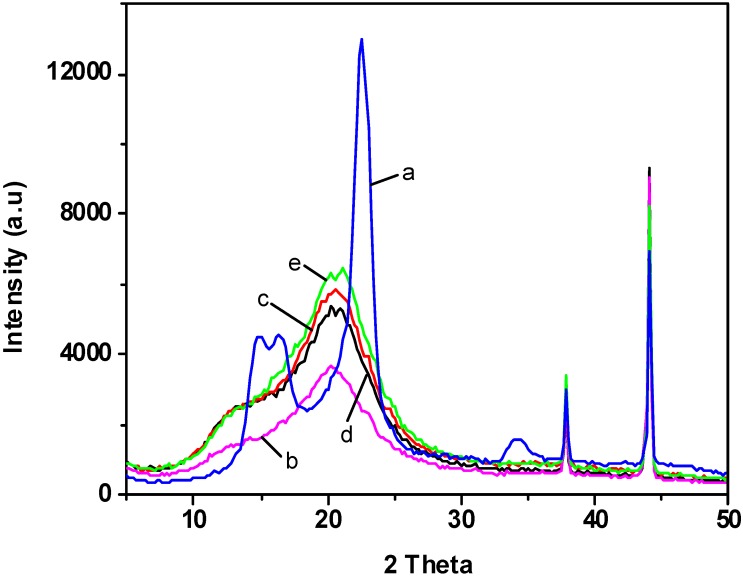
X-ray diffraction (XRD) images of samples: (**a**) cotton linter; (**b**) control sample; (**c**) cellulose film plasticized with sorbitol; (**d**) cellulose film plasticized with glycerol; (**e**) cellulose film plasticized with CMC.

The X-ray patterns of cotton linter and different plasticized cellulose films are shown in Figure 4. The diffraction peaks from cotton linter at 2*θ* = 14.67°, 16.39° and 22.53° for (110), ($1\bar{1}0$) and (200) planes are characteristic for cellulose I crystal [34], and those from regenerated cellulose films with and without plasticizers at 2*θ* = 12.1°, 19.8° and 22.0° for ($1\bar{1}0$), (110) and (200) planes are assigned to cellulose II crystal [35]. Cellulose II can be obtained by recrystallization of native cellulose, which indicated that the crystalline structure of the regenerated cellulose was converted into cellulose II during the dissolving and regenerating process. It has been found that the hydrogen bonds in cellulose I exist only between chains belonging to the same sheet [36]. However, the hydrogen bonds in cellulose II are also found between sheets, which means they form a three-dimensional (3D) network [37]. Therefore, cellulose II is considered as the most stable structure of technical relevance compared with other crystal structures [38]. The crystallinity index of the regenerated cellulose films were around 38%, which were lower than that of the control cellulose film (54%). It has been shown that the regenerated cellulose exhibited lower crystallinity degrees compared with native cellulose when being dissolved in ionic liquids [39], which was probably caused by the rapid depolymerization of cellulose in the dissolving process [19].

### 2.5. Thermogravimetric Analysis

**Figure 5:**
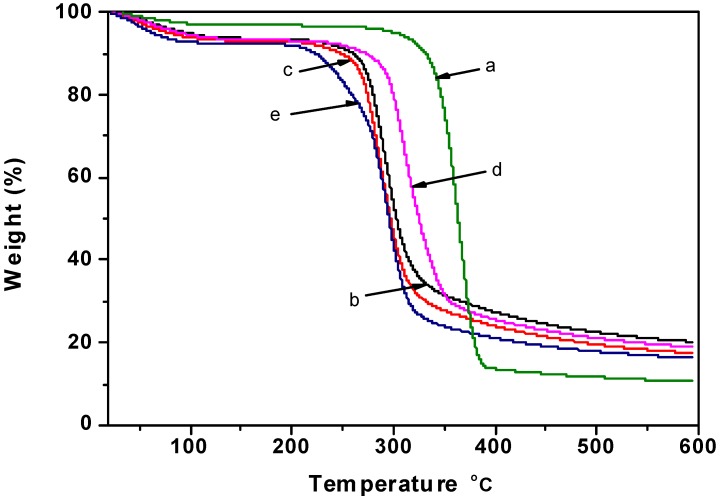
The thermogravimetric analysis (TG) curves of samples: (**a**) cotton linter; (**b**) control sample; (**c**) cellulose film plasticized with sorbitol; (**d**) cellulose film plasticized with glycerol; (**e**) cellulose film plasticized with CMC.

**Figure 6:**
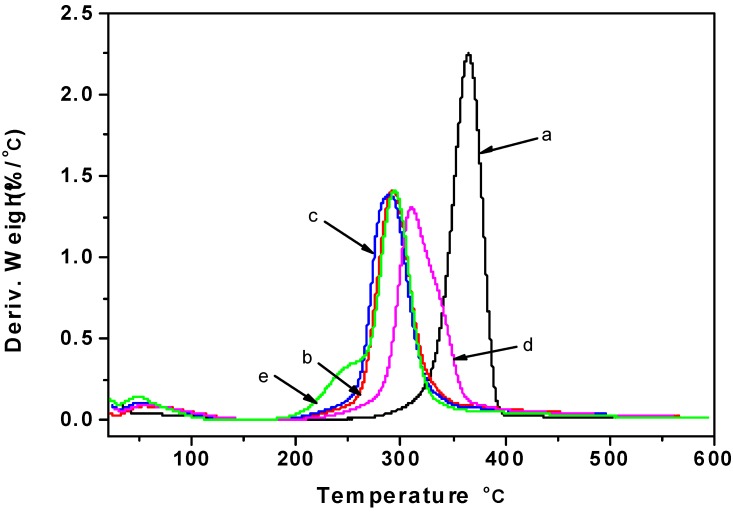
The differential thermal analysis (DTA) curves of samples: (**a**) cotton linter; (**b**) control sample; (**c**) cellulose film plasticized with sorbitol; (**d**) cellulose film plasticized with glycerol; (**e**) cellulose film plasticized with CMC.

The effect of blending plasticizers on the thermal stability of regenerated cellulose films was investigated by thermal degradation, and the thermogravimetric analysis (TG) and differential thermal analysis (DTA) curves are shown in Figure 5 and Figure 6. Cellulose is a polymer with moderate thermal stability, and rapid chemical decomposition occurs between 315 and 400 °C [40]. As seen from Figure 5, the thermal stability of cotton linter was higher than that of regenerated cellulose films. It was noteworthy that the cellulose films plasticized with glycerol showed a higher thermal stability compared with other cellulose films. As shown from the DTA curves, the pyrolysis of film plasticized with CMC showed two well-resolved degradation peaks: the first weight loss peak (240 °C) was attributed to the thermal decomposition of the CMC segments, while the second major weight loss peak (290 °C) was assigned to the thermal decomposition of the cellulose chains.

### 2.6. ^13^C CP/MAS NMR Spectra

**Figure 7:**
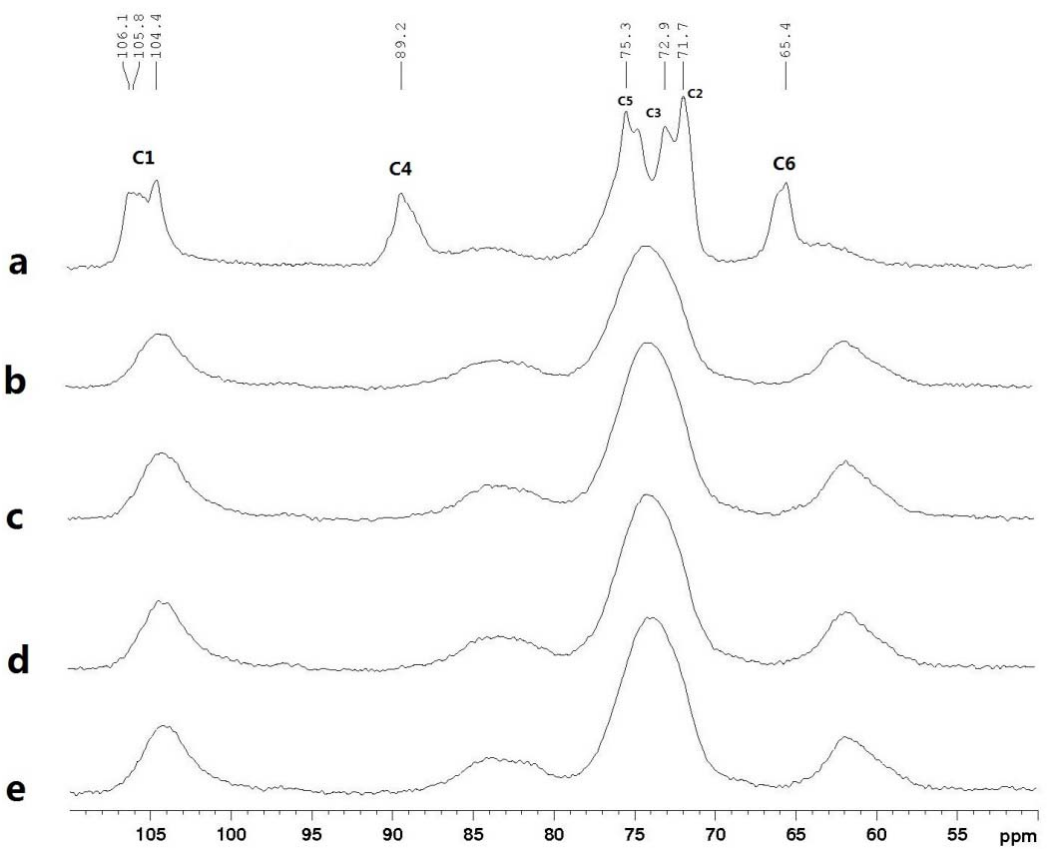
The ^13^C solid-state cross-polarization/magic angle spinning nuclear magnetic resonance (CP/MAS NMR) spectra: (**a**) cotton linter; (**b**) control cellulose film; (**c**) cellulose film plasticized with sorbitol; (**d**) cellulose film plasticized with glycerol; (**e**) cellulose film plasticized with CMC.

The ^13^C CP/MAS (cross-polarization/magic angle spinning) spectra for the cotton linter (sample a) and different plasticized cellulose films (sample b, c, d, e) are shown in Figure 7. The main peaks of cotton linter were the superimposed C-2, C-3 and C-5 signals at 71.7, 72.9 and 75.3 ppm, and other prominent features were the cellulose C-1 (105.8 ppm), C-4 (89.2 ppm) and C-6 (65.4 ppm), which indicated that the crystalline structure of cotton linter is typical of cellulose I [41]. The signals of cotton linter at 89.2 ppm and 84 ppm (data not shown) are due to the crystalline and amorphous regions (C-4), respectively. It was noted that the peak attributed to crystalline cellulose at 89.2 ppm (C-4) in regenerated cellulose films (spectra b–e) almost disappeared compared with cotton linter (spectrum a). This indicated that the crystallinity index of regenerated cellulose dramatically decreased, due to the dissolving treatment, which was consistent with previous study [42]. It has been shown that the C-6 resonance occurs as a singlet near 63 ppm in the cellulose II [37]. Thence, the only peak in regenerated cellulose films near 62 ppm indicated that the transition from cellulose I to II had taken place during preparation. 

### 2.7. Mechanical Properties 

**Figure 8:**
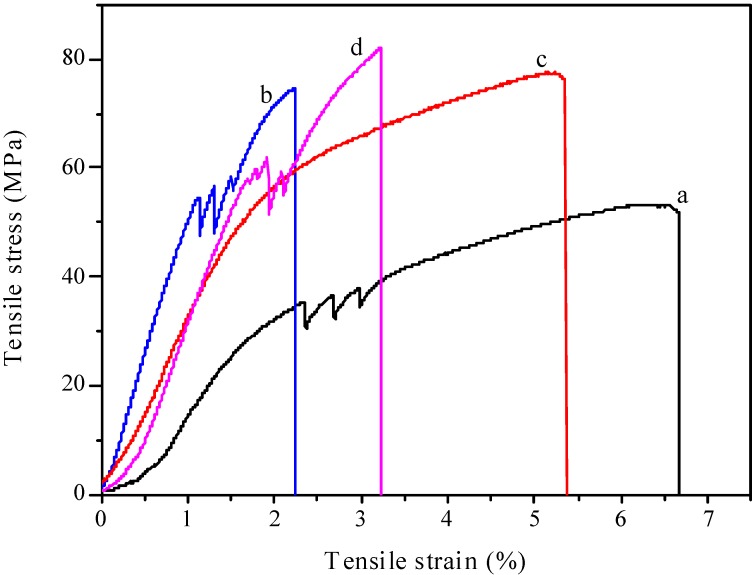
The tensile-strain curves of the films: (**a**) control sample; (**b**) cellulose film plasticized with sorbitol; (**c**) cellulose film plasticized with glycerol; (**d**) cellulose film plasticized with CMC.

The stress-strain curve of the controlled cellulose film (curve a) and all plasticized cellulose films (curves b, c, d) are shown in Figure 8. It was noted that the tensile strength of the control cellulose film was relatively weak. In comparison with the control sample, the tensile stress of the plasticized cellulose films was increased by 43.5%, 46.2% and 58% with the addition of sorbitol, glycerol and CMC, respectively. The interface between the cellulose matrix and plasticizers play an important role in the improvement of the mechanical properties of the cellulose films. Therefore, the strengthening of the cellulose films might be due to the formation of hydrogen bonds between the cellulose matrix and the plasticizers [43]. Moreover, van der Waals forces also led to good interfacial adhesion between cellulose matrix and the plasticizers [28]. Therefore, the increases of tensile stress are probably caused by the formations of new hydrogen bonds networks and stacking interactions between cellulose and the different plasticizers. In addition, it can be observed that all the cellulose films showed thermoplastic-like behavior, with stress increasing rapidly at small strains and more slowly after a yield point.

## 3. Experimental Section 

### 3.1. Materials

Cotton linter fiber with the degree of polymerization (DP) 920 was kindly supplied by Silver Hawk Fiber Corporation (Shandong province, China). All chemicals were analytical grade reagents and used as received without further purification. Ionic liquid, 1-allyl-3-methylimidazolium chloride (AmimCl), with purity of ≥95%, was purchased from Lanzhou Institute of Chemical Physics.

### 3.2. Preparation of Regenerated Cellulose Film

A typical experimental procedure for preparation of regenerated cellulose films with ionic liquids was as follows. First, the cotton fiber (5% weight of the ionic liquid) was dissolved in ionic liquid AmimCl under magnetic stirring for 30 min at 90 °C. Then, plasticizers (25% weight of the cotton fiber) were added in cellulose/AmimCl mixture with magnetic stirring for 30 min at 90 °C. The mixture was degassed by being kept standing at 90 °C for 1 h. After that, the cellulose films were prepared by casting the homogeneous mixture on glass plate. Then, the cellulose films were soaked in deionized water and washed thoroughly to remove ionic liquid. The obtained cellulose films were dried in atmosphere for 48 h and stored in moisture controlled desiccators. 

### 3.3. Characterization 

#### 3.3.1. Thickness

The thickness of regenerated cellulose film was measured by a micrometer (Lorentzen & Wettre, precision 1 μm). The thickness of six different locations on each film was detected, and the average values were used in the calculation of the mechanical test.

#### 3.3.2. FTIR Spectra

FTIR spectra were measured using a Tensor 27 infrared spectrum instrument (Bruker, German). The scan range was 400–4000 cm^−1^, and the resolution rate was 2 cm^−1^.

#### 3.3.3. Scanning Electron Microscopy

A scanning electron microscopy (SEM) instrument (S-3400N, Hitachi, Japan) was used to observe the surfaces of regenerated cellulose films at an accelerating voltage of 10 kV. The surface was coated with gold by a sputter coater (E-1010, Hitachi, Japan) before detecting.

#### 3.3.4. Atomic Force Microscopy

The morphology of the film surface was also studied by atomic force microscopy (AFM) (SPM-9600, Shimadzu, Japan). Small pieces of films were glued onto metal disks and attached to a magnetic sample holder located on the top of the scanner tube. Phase images were recorded under ambient air conditions. All of the images were recorded in contact mode in air using silicon cantilevers.

#### 3.3.5. X-ray Diffraction

The X-ray diffraction (XRD-6000, Shimidzu, Japan) method was used to determine the crystallinity of the regenerated cellulose film samples. The X-ray diffractograms were record in reflection mode in the angular range of 5°–40° (2*θ*) with a scanning speed of 5°/min. 

#### 3.3.6. Thermogravimetric Analysis

Thermogravimetric analysis (TG) and differential thermal analysis (DTA) were carried out on a Q600SDT instrument (TA, USA). The samples weighing around 10 mg were detected from room temperature to 600 °C under nitrogen flow with a heating rate 10 °C/min.

#### 3.3.7. ^13^C CP/MAS NMR Spectra

Solid-state cross-polarization/magic angle spinning (CP/MAS) ^13^C NMR spectra of the regenerated cellulose film samples were obtained at 100.6 MHz using a Bruker AV-III 400 M spectrometer (Germany). The cellulose films was packed in a 4 mm zirconia (ZrO_2_) rotor, and the measurement was performed using a CP pulse program with a 2 ms contact time and a 2 s delay between transitions. The spinning rate was 5 kHz.

#### 3.3.8. Tensile Strength Test 

Tensile testing was performed on a Zwick/Z005 (Zwick/Roell, Germany) instrument fitted with a 200 N load cell with the crosshead speed of 0.5 mm/min, and the initial distance between the grips was 20 mm. Eight specimens of each formulation were tested, and the average values were calculated. The measurements were performed under room temperature.

## 4. Conclusions 

In this paper, regenerated cellulose films with enhanced mechanical properties were successfully prepared by incorporating different plasticizers using ionic liquid 1-allyl-3-methylimidazolium chloride (AmimCl) as the solvent. SEM and AFM images showed that the surface of the regenerated cellulose films displayed homogeneous and smooth morphology. XRD suggested that the crystalline structure of the regenerated cellulose was transferred from cellulose I to cellulose II after regeneration. The regenerated cellulose film plasticized with glycerol exhibited better thermal stability compared with other cellulose films. Incorporation of plasticizers resulted in better film formation and a significant improvement in the tensile strength. These results displayed an effective and environmentally friendly method to prepare regenerated cellulose film with high quality. 
